# Assessing Cognitive Deterioration After COVID-19 Infection (The ACDC Study): An Exploratory Multimodal Neuroimaging Study

**DOI:** 10.3390/jcm15114241

**Published:** 2026-05-30

**Authors:** Jonathan McLaughlin, Gordon Waiter

**Affiliations:** 1 Department of Psychological Medicine, NHS Grampian, Ashgrove House, Aberdeen Royal Infirmary, Aberdeen AB25 2ZN, UK; 2 Aberdeen Biomedical Imaging Centre, University of Aberdeen, Foresterhill, Aberdeen AB25 2ZD, UK

**Keywords:** post-COVID-19 condition, cognitive impairment, magnetic resonance spectroscopy, functional cognitive disorder, functional neurological disorder, neuroimaging biomarkers

## Abstract

**Background:** Cognitive difficulties are common after SARS-CoV-2 infection, yet their neurobiological underpinnings remain uncertain. Cognitive symptoms in post-COVID-19 condition (PCC) are often characterised by attentional and executive dysfunction, although the relationship between subjective symptoms and objective neurobiological changes remains uncertain. **Methods:** Adults previously hospitalised with COVID-19 who reported persistent cognitive symptoms underwent neuropsychological testing and 3 T MRI. The protocol included high-resolution volumetric imaging, diffusion-based tractography, and magnetic resonance spectroscopy (MRS) of frontal white matter. Data were compared with age- and sex-matched controls from a pre-COVID-19 cohort and against pooled normative MRS datasets. Analyses adjusted for intracranial volume, sex, and time since infection, with false-discovery-rate correction. This study was exploratory and hypothesis-generating in design. **Results:** Thirty participants were recruited; twenty-nine completed MRI acquisition. Participants (mean age 58 years; 62% female; approximately two years post-infection) demonstrated selective impairments in attention, working memory, and verbal fluency. No widespread volumetric or white-matter differences were identified, although reduced posterior hypothalamic volume and altered occipito-parietal connectivity were observed. MRS demonstrated reduced N-acetylaspartate and elevated choline, myo-inositol, and glutamate-glutamine ratios relative to normative reference ranges. No significant associations were observed between imaging measures and cognitive or symptoms outcomes after correction. **Conclusions:** PCC is characterised by circumscribed cognitive changes and subtle neural differences, but these objective changes do not closely align with subjective symptom severity. This mismatch shares phenotypic features with functional cognitive disorder and suggests that post-COVID-19 “brain fog” is not driven by structural or neurochemical changes alone. Instead, it potentially reflects a combination of mild neurobiological effects and functional cognitive processes. Together, these findings highlight the importance of considering both brain-based and functional contributors to persistent cognitive complaints after SARS-CoV-2 infection.

## 1. Introduction

Cognitive dysfunction following SARS-CoV-2 infection is commonly reported. In one hospitalised cohort, 59% and 53% reported memory and concentration problems, respectively, at three years’ follow up [1]. Additionally, in a large meta-analysis of hospitalised and community subjects who had at least six months of follow-up, cognitive impairment and concentration impairment had estimated prevalences of 27.1% and 23.8%, respectively [2].

A large study in the UK population, albeit based on subjective reporting, found persistent cognitive impairment equivalent to minus three IQ points on a global measure of cognition in subjects with mild COVID-19 infection [3]. This increased to minus six and minus nine points, respectively, in subjects with persistent COVID-19 symptoms and in those who had been admitted to ITU owing to infection.

In several different studies, the cognitive dysfunction has been characterised as impairment in executive function, attention, memory and processing speed [4,5,6]. Various aetiological mechanisms have been discussed in relation to these findings, including glial cell activation perpetuating neural pathology, endotheliopathy with microvascular disruption and blood–brain-barrier breakdown, and autoimmunity [7,8].

Neuroimaging data to substantiate such processes are mixed, while suggestive. In a large-scale analysis using the UK Biobank [9], modest reductions in grey matter were reported in the orbitofrontal cortex and parahippocampal gyrus, as well as reduced global brain volume, when compared with pre-pandemic control subject data. Other studies have found no structural changes [10], while further work reported the absence of significant volumetric change but reported brain region connectivity changes potentially applicable to cognitive attentional problems [11]. A recent meta-analysis of 78 studies highlighted the mixed evidence of grey matter change, white matter integrity, cortical thickness alteration and functional connectivity findings [12].

Given the proposed aetiological processes, magnetic resonance spectroscopy (MRS) is an advanced MRI methodology that could offer insights into the pathogenesis of reported cognitive change following SARS-CoV-2 infection. Early work during the pandemic in a series of three patients with COVID-19-associated leukoencephalopathy suggested decreased N-acetylaspartate (NAA), with the authors suggesting this might reflect neuronal dysfunction and axonal injury [13]. Two further studies corroborated this finding [14,15] but did not report the significant increases in a wider group of metabolites from the original work; Choline (Cho), Myo-inositol (mI), Glutamate–Glutamine (Glx) and Lactate (Lac). A further study found no significant change [16], although the specific brain region studied was different. See Table 1 for a comparison of these findings across studies.

In general, these studies are heterogenous in the methodologies employed, and the results are mixed. Individual studies look at different brain regions, different time periods since primary infection, and severities of COVID-19 disease, and some use control comparators while others do not. Other work sees the use of different methods of reporting metabolite concentrations or a focus on non-standard MRS metabolites [18].

Although cognitive changes in post-COVID-19 condition (PCC) may resemble those seen in functional cognitive disorders (FCDs) [19]—characterised by persistent cognitive complaints without clear neuropathology and with inconsistencies between subjective reports and objective findings—this study sought to clarify whether, like FCD, PCC “brain fog” reflects anatomical or neural correlates detectable by advanced MRI. We adopted an exploratory multimodal framework to characterise potential structural, connectivity, and neurochemical features associated with cognitive difficulties. We also examined whether any imaging alterations were related to individual differences in neuropsychological performance. Based on the prior mixed evidence, we anticipated that any abnormalities would be subtle and that imaging–cognition associations might be modest.

## 2. Materials and Methods

### 2.1. Participants

Participants were recruited through the Mental Health After COVID-19 Hospitalisation (MACH) service in NHS Grampian. The MACH service was a Scottish Government initiative to assess and manage the mental health needs of those hospitalised due to the SARS-CoV-2 virus. Eligible individuals had been hospitalised with confirmed SARS-CoV-2 infection (PCR positive) and subsequently reported cognitive difficulties. Additional inclusion criteria were age ≥ 18 years, ability to provide informed consent, and willingness to undertake neuropsychological assessment. Exclusion criteria included prior intensive care admission for COVID-19 (to reduce heterogeneity in illness severity), pre-existing neurodegenerative disease, moderate/severe brain injury, neuroinflammatory disorder (e.g., multiple sclerosis), history of cognitive impairment predating hospitalisation, or MRI contraindications. All participants provided written informed consent. The study was approved by the appropriate NHS Research Ethics Committee and conducted in accordance with the Declaration of Helsinki. As this was an exploratory pilot study designed to characterise multimodal effects rather than test a single primary end point, no formal power calculation was performed.

A total of 30 participants were recruited. One participant (female) discontinued MRI acquisition part-way through the protocol after completing four sequences and was excluded from imaging analyses but retained for screening and neuropsychological analyses. Accordingly, imaging analyses were conducted on n = 29 participants, while screening and cognitive data included *n* = 30, with variable completion across specific neuropsychological tests.

### 2.2. Screening and Neuropsychological Assessment

As part of standard care within MACH, all participants completed validated screening measures: EQ-5D-5L (health-related quality of life), FACIT-Fatigue (v4), Cognitive Change Index (CCI-20-S), Generalised Anxiety Disorder scale (GAD-7), Patient Health Questionnaire (PHQ-9), CORE-10, and Trauma Screening Questionnaire (TSQ). These measures were included to characterise the broader clinical and psychological profile of the cohort and to examine whether symptom burden across domains of mood, fatigue, perceived cognitive change, and psychological distress was associated with objective neuropsychological performance or neuroimaging outcomes—given well-established relationships between affective symptoms, fatigue, and cognition, and the high prevalence of these symptoms in PCC.

Formal neuropsychological testing was conducted in those reporting cognitive symptoms and consenting to take part in the study. The battery was designed to sample key cognitive domains implicated in PCC; attention, executive function, working memory, and episodic memory. Tests included Wechsler Test of Adult Reading (WTAR, premorbid functioning), California Verbal Learning Test-III (CVLT-3), Logical Memory subtest of the Wechsler Memory Scale-IV, Rey–Osterrieth Complex Figure Test (ROCFT), Digit Span Forward and Backward (WMS-IV), Elevator Counting and Elevator Counting with Distraction (Test of Everyday Attention), Verbal Fluency (Delis–Kaplan Executive Function System), Hayling and Brixton Tests, Zoo Map (Behavioural Assessment of the Dysexecutive Syndrome), and the Colour Trails Test. Standardised z-scores were derived from published norms, and impairment was defined as performance ≥ 1.5 SD below age-adjusted normative means. Neuropsychological test performance was not compared with the STRADL cohort, as that study was not designed as a matched cognitive comparator for a clinically selected PCC population; instead, published normative datasets provided the reference standard for neuropsychological interpretation, as described above.

Tests were grouped by the following cognitive domains: premorbid intellectual functioning was estimated using the WTAR; episodic verbal memory was assessed via the CVLT-3 (list learning, recall, and recognition) and Logical Memory (story recall); visuospatial memory and constructional ability were assessed via the ROCFT (copy, immediate and delayed recall); working memory was assessed using Digit Span Forward (short-term verbal retention) and Backward (manipulation of held information); sustained attention and attentional control were assessed using the TEA Elevator Counting (sustained attention) and Elevator Counting with Distraction (divided attention) subtests; executive function and verbal retrieval were assessed using Verbal Fluency (letter and category), Hayling (response initiation and suppression), Brixton (rule detection and cognitive flexibility), and Zoo Map (planning and strategy); and processing speed and set-shifting were assessed using the Colour Trails Test.

### 2.3. MRI Acquisition

Imaging was performed on a Philips Ingenia dStream 3T scanner (Software version R5.3) at the Aberdeen Biomedical Imaging Centre. The protocol comprised high-resolution 3D T1-weighted imaging, T2-weighted imaging, fluid-attenuated inversion recovery (FLAIR), magnetic resonance spectroscopy (MRS), diffusion tensor imaging (DTI) and susceptibility-weighted imaging with quantitative susceptibility mapping (QSM). White matter hyperintensity lesion load was derived from FLAIR. Diffusion metrics enabled tractography and structural connectivity analyses. Resting-state data were used for network-based statistics. All scans underwent quality control assessment, including visual inspection for motion artefact, FreeSurfer version 7.1.1 reconstruction quality control, and verification of spectroscopy voxel placement.

### 2.4. Volumetric Analysis

Regional cortical volumes were compared with age- and sex-matched controls drawn from the Stratifying Resilience and Depression Longitudinally (STRADL) study [20], acquired on the same scanner platform. The STRADL study is a depression-focused neuroimaging investigation of Generation Scotland participants, incorporating detailed clinical, cognitive, and neuroimaging assessments conducted at 3 T (Philips Ingenia dStream, Aberdeen Biomedical Imaging Centre); full cohort details are reported by Habota et al. Age- and sex-matched controls were selected from this pre-COVID-19 cohort for all aspects of the imaging protocol except MRS comparison. Volumes were extracted and reprocessed using FreeSurfer version 7.1.1 for quality control and adjusted for estimated total intracranial volume (eTIV).

### 2.5. Magnetic Resonance Spectroscopy

**Figure 1:**
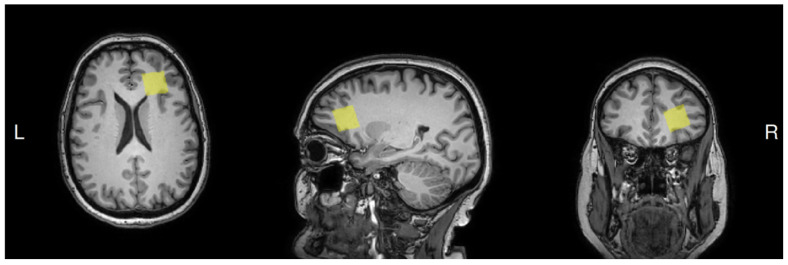
Yellow square shows the voxel location in frontal white matter for magnetic resonance spectroscopy.

Single-voxel point-resolved spectroscopy (PRESS) was acquired from a pre-specified region in the frontal white matter (FWM) (see Figure 1), previously implicated in COVID-19-related cognitive dysfunction [13,15]. Spectra were processed using jMRUI software, applying the Adaptive Modelling Algorithm for Resonance Spectroscopy (AMARES) for peak fitting. Metabolite ratios were expressed relative to creatine (Cr): N-acetylaspartate (NAA/Cr), choline (Cho/Cr), myo-inositol (mI/Cr), and glutamate–glutamine complex (Glx/Cr).

To generate normative metabolite-to-creatine ratios (/Cr) in frontal white matter at 3 T, we pooled two healthy adult datasets. The first [15] included 25 controls studied with single-voxel PRESS, from which we derived/Cr ratios by dividing mean metabolite concentrations (tNAA, Cho, tCr, mI, Glx) by mean tCr. The second [21] comprised 125 adults scanned with whole-brain short-TE Magnetic resonance spectroscopic imaging (MRSI), providing frontal and sublobar white matter/Cr ratios. These datasets were acquired using comparable field strength (3 T) but differed in acquisition protocols and processing pipelines.

We combined study means using inverse-variance weighting to obtain pooled estimates and Standard Errors for NAA/Cr, Cho/Cr, mI/Cr, and Glx/Cr, which served as normative reference ranges for patient comparisons. Although normative datasets were acquired using similar MRS protocols, cross-cohort variation in acquisition parameters may introduce systematic differences. Consequently, pooled reference values should be interpreted as approximate reference ranges and MRS findings should be interpreted as indicative of potential neurometabolic differences rather than definitive group-level effects.

### 2.6. Statistical Analysis

Analyses were conducted in R. Demographic and clinical characteristics were summarised descriptively. Group-level volumetric comparisons between participants and STRADL controls were performed using linear models, adjusting for eTIV, age and sex. White matter hyperintensity burden and lesion counts were compared using independent-samples *t*-tests. Network-based statistics were applied to identify connectivity differences between groups, with Benjamini–Yekutieli false discovery rate (BY-FDR) correction. BY-FDR was selected as it robustly controls false discovery rate in the context of dependence amongst neuroimaging parameters.

For spectroscopy, mean metabolite ratios were compared against normative reference values using one-sample *t*-tests, adjusting for time from infection to scan. Multiple-comparison control was applied using the BY-FDR procedure. Associations between imaging measures (volumetric, connectivity, spectroscopy) and cognitive/symptom outcomes were tested using general linear models, adjusting for age, sex, and time since infection. Covariates were chosen based on known associations between neuroimaging and cognitive outcomes. Screening measure-cognition associations were examined similarly. Exploratory associations not surviving correction were reported as nominal findings only. Statistical significance was defined at *p* < 0.05 after correction.

## 3. Results

### 3.1. Demographic, Clinical and Neuropsychological Characteristics

**Figure 2:**
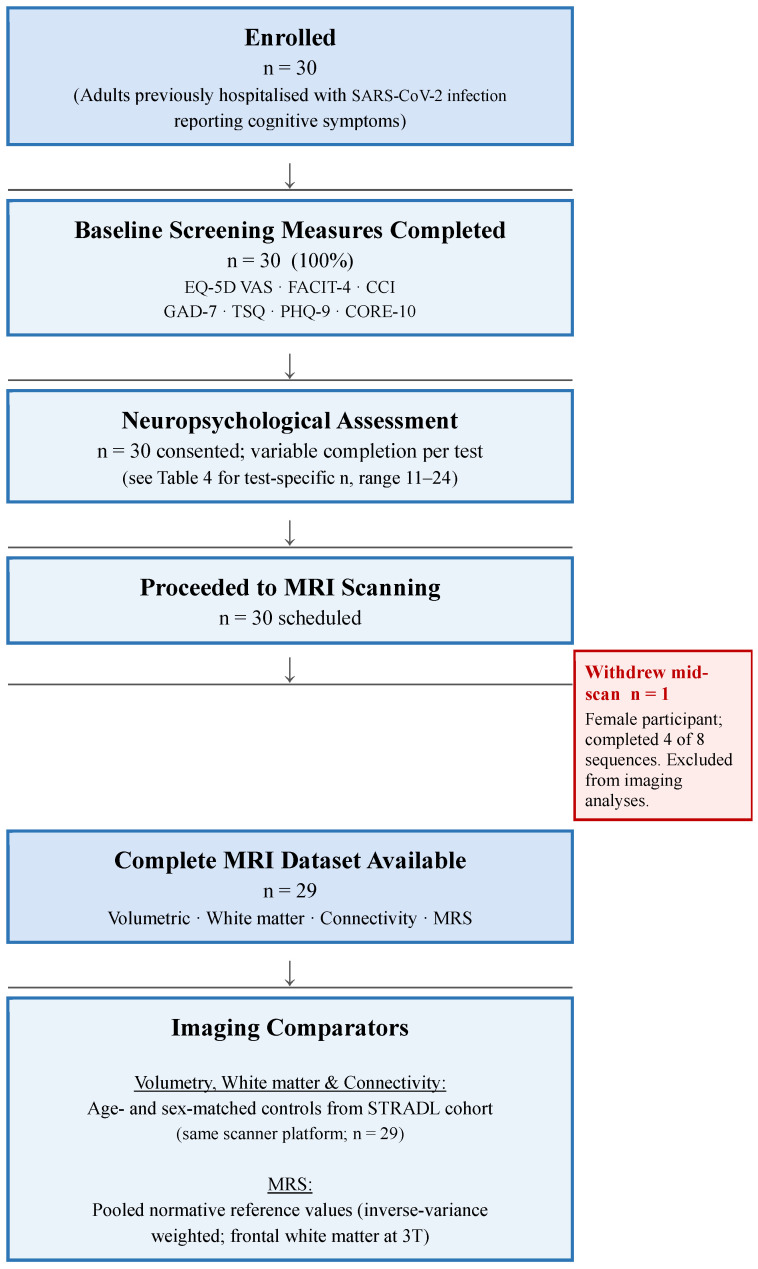
ACDC Study—Participant Flow Diagram. Participant numbers for individual neuropsychological tests vary due to test-specific eligibility/selection and participant fatigue; see Table 3 for complete breakdown. The participant who withdrew mid-scan completed all baseline screening and neuropsychological measures and is included in those analyses (n = 30). ACDC = Assessing Cognitive Deterioration after COVID-19.

Participant demographics are summarised in Table 2. Participant flow and analysis inclusion are summarised in Figure 2. Imaging analyses were conducted on n = 29 participants following exclusion of one incomplete scan, while screening measures included n = 30. Neuropsychological test completion varied by measure (range n = 11–24), as detailed in Table 3.

Table 4 presents summary scores from baseline screening questionnaires. Overall, participants reported variable health-related quality of life with moderate levels of fatigue (FACIT-4) and perceived cognitive change (CCI). Scores on measures of anxiety (GAD-7) and depression (PHQ-9) indicated moderate symptom burden in the cohort.

Neuropsychological assessment (Table 3) identified the most consistent weaknesses in attention and working memory, with reduced performance on the TEA Elevator Counting with Distraction task (z = −0.93; 30% impaired) and Digit Span Forward (z = −1.12; 33%) and Backward (z = −1.03; 24%). Verbal fluency was also affected, particularly on the category task (z = −0.79; 30%), relative to letter fluency (z = −0.47; 19%). In contrast, performance on the Trail Making Test (Parts A and B), verbal memory (CVLT), and premorbid functioning (WTAR) was generally within expected ranges.

This pattern suggests particular difficulties with attentional control, verbal retrieval, and short-term memory processes.

### 3.2. Volumetric Analysis

Regional cortical volume comparisons between the ACDC participants and age-matched controls from the Stratifying Resilience and Depression Longitudinally (STRADL) cohort (Supplementary Table S1—Supplementary Materials) revealed no statistically significant differences after correction for multiple comparisons. While some regions showed nominal group differences in raw *p*-values (e.g., in the inferioparietal volume bilaterally and right entorhinal volume), none survived false discovery rate (BY-FDR) adjustment. These findings suggest that, at the group level, there were no robust volumetric alterations detectable across cortical regions in the ACDC cohort relative to controls. All FreeSurfer version 7.1.1 reconstructions passed visual quality control, reducing the likelihood that null effects were due to segmentation artefacts.

Across the 12 hypothalamic subregions, only the left posterior hypothalamus showed a significant between-group difference after adjusting for sex, eTIV (Table 5) and BY-FDR correction. In this adjusted model, the ACDC group had a smaller left posterior volume than controls (group difference = −12.45 mm^3^, 95% CI = −19.78 to −5.11, *p* = 0.001; BY-FDR *p* = 0.047). This isolated finding should be interpreted cautiously pending replication. No other hypothalamic region remained significant after BY-FDR correction.

### 3.3. White Matter

Group comparisons of white matter lesion metrics revealed no significant differences between the ACDC and control group (Supplementary Table S2—Supplementary Materials). Mean total lesion volume was comparable across groups (ACDC: 2.89 mL, Controls: 3.13 mL; *p* = 0.85), as was the mean number of lesions (ACDC: 7.71, Controls: 7.39; *p* = 0.77).

### 3.4. Connectivity

Network-based statistics identified a significant cluster of connections (Cluster 4/171) (see Table 6) showing group differences (*F*(2,51) = 8.01, *p* = 0.00094, FDR-corrected *p* = 0.030). This cluster comprised bilateral connections between the lateral occipital cortex (superior division; sLOC) and the superior parietal lobule (SPL).

Two connections remained significant after FDR correction: the right lateral occipital cortex (superior; sLOC r) to left superior parietal lobule (SPL l) connection (*T*(52) = 3.66, *p* = 0.00059, FDR *p* = 0.0176) and the left lateral occipital cortex (superior; sLOC l) to right superior parietal lobule (SPL r) connection (*T*(52) = 3.54, *p* = 0.00085, FDR *p* = 0.0192).

### 3.5. Magnetic Resonance Spectroscopy

When compared with computed normative reference values, the cohort demonstrated a distinct pattern of neurometabolic alterations (Table 7). Mean Cho/Cr, Glx/Cr, and mIns/Cr ratios were elevated, whereas NAA/Cr was reduced. For all metabolites, the 95% confidence intervals for the adjusted means did not overlap with the corresponding normative values, indicating consistent group-level differences.

Statistical analyses, controlling for days from positive COVID-19 test to scan date, and employing BY-FDR to account for multiple comparisons, confirmed that all observed deviations from normative values were highly significant (all adjusted *p* < 0.05).

### 3.6. Cross-Domain Correlation Analyses

Across all models, no significant associations were observed between MRS metabolite ratios (NAA, Cho, Glx, mI) and cognitive or screening outcomes when adjusting for age, sex, and days since scanning. Likewise, analyses of hypothalamic subregional and whole-volume measures (left/right, anterior/posterior subdivisions) corrected for eTIV did not reveal significant associations with cognitive or screening scores. Although some uncorrected models produced nominal effects, none survived BY-FDR correction for multiple comparisons. No hypothalamic volumetric measures showed nominal associations with cognitive or screening outcomes.

### 3.7. Screening Measures and Cognitive Outcomes

In examining associations between screening measures and cognitive performance while adjusting for age and sex, no associations survived BY-FDR. At the unadjusted level, several screening measures showed nominal associations with cognitive outcomes. The CCI, PHQ-9, and FACIT-4 appeared most frequently, while verbal fluency tasks—particularly letter phonemic fluency—along with Rey immediate and delayed recall, were the cognitive measures most often implicated. This suggests a tentative pattern in which higher symptom burdens in perceived cognitive change, depression symptoms and perceived fatigue were linked to poorer fluency and memory performance; however, as none of these effects survived correction for multiple comparisons, they should be considered exploratory. Associations between screening measures and neuroimaging outcomes (volumetric, connectivity, and MRS measures) were examined as part of the cross-domain correlation analyses, also adjusting for age and sex; no associations survived BY-FDR correction in those models either. Adequately powered analyses examining age and sex as moderators of screening–cognition and screening–imaging relationships were not feasible in this exploratory sample and represent a target for future larger-scale studies.

## 4. Discussion

This exploratory multimodal neuroimaging study of individuals with PCC identified selective impairments in attention, working memory, and verbal fluency, accompanied by consistent neurometabolic alterations but little evidence for widespread structural change. Although a reduction was observed in posterior hypothalamic volume, and connectivity differences were detected in occipitoparietal circuits, these effects were limited and not robustly correlated with neuropsychological performance. These findings suggest that while subtle neural alterations persist up to two years after infection, their contribution to the subjective experience of “brain fog” remains uncertain.

The neuropsychological profile observed here is consistent with previous reports of post-COVID-19 cognitive sequelae [12,22]. Deficits were most marked in executive and attentional domains, while global cognition and episodic memory were relatively preserved, echoing other studies [23]. Such circumscribed impairments correspond closely with patients’ descriptions of attentional lapses and reduced fluency.

Unlike the UK Biobank analysis [9], which identified small but significant reductions in orbitofrontal and parahippocampal regions and global brain volume loss, we observed no widespread volumetric differences. This might reflect cohort composition, timing post-infection or the absence of pre-infection imaging in the same subjects.

Other studies have reported cortical thinning, subcortical structure changes and altered white matter integrity [24,25,26], though null findings are also common. Our largely negative volumetric results therefore align with this mixed literature and emphasise how differences in cohort characteristics, imaging protocols, and follow-up intervals shape outcomes.

Although speculative, the posterior hypothalamic volume reduction we observed is noteworthy given the role of the hypothalamus in neuroendocrine regulation and fatigue [27]. In considering a mechanistic link, it is notable that the tuberomammillary nucleus (TMN) lies within the posterior hypothalamus. The TMN is the brain’s primary source of histaminergic neurons and plays a central role in regulating wakefulness, arousal, and attention [28]. More broadly, the posterior hypothalamus contributes to autonomic regulation and neuroendocrine control, supporting homeostatic balance under stress and influencing cardiovascular and thermoregulatory function [29]. Thus, COVID-19-related structural change in this region could plausibly disrupt histaminergic and hypothalamic circuits, contributing to hallmark features of post-COVID-19 condition (PCC) such as fatigue, dysautonomia, and cognitive disturbance. This raises the possibility of a pathophysiological connection, though replication is required given the singularity of this finding, and the interpretation outlined above should be viewed as hypothesis-generating.

White matter lesion burden was similar between groups, indicating conventional small-vessel pathology is unlikely to underlie symptoms. More subtle changes in network organisation may be relevant. We observed bilateral occipitoparietal connectivity alterations involving the superior lateral occipital cortex and superior parietal lobule. These occipitoparietal pathways support attentional control, and the superior parietal lobule is seen as an intrinsic part of the dorsal attention network (DAN) [30,31]. These findings provide a plausible neural correlate for the issues in attentional domains reported by subjects and observed in the neuropsychological profile.

Reduced structural connectivity within this circuit is consistent with impaired top-down attentional modulation, which may underlie the observed deficits in sustained attention and working memory evident in the neuropsychological profile—particularly the performance decrements on the TEA Elevator Counting with Distraction and Digit Span tasks. The imaging protocol was not designed to map individual modalities to specific cognitive domains a priori; rather, the multimodal approach was designed to test the hypothesis that there would be neural correlates of reported cognitive symptoms in general.

By contrast, Carreras-Vidal et al. [32] reported widespread intra- and inter-network connectivity increases linked to cognition and symptom measures. Our more focal findings may reflect methodological differences, sample size, or attenuation of global effects over time, leaving residual alterations in attentional circuits.

MRS provided the clearest biological signal. Elevated Cho/Cr, mI/Cr, and Glx/Cr ratios, alongside reduced NAA/Cr, were robust after multiple-comparison correction. Reduced NAA typically reflects neuronal dysfunction [33], while increased Cho and mI suggest membrane turnover and glial activation [34]. Alterations in Glx are widely considered abnormal, as they reflect disrupted glutamatergic metabolism. Elevated Glx has been associated with excitotoxic stress and neuroinflammation, while reduced Glx suggests impaired excitatory neurotransmission or altered signalling [15,35]. This profile is consistent with proposed mechanisms of neuroinflammation and altered glutamatergic signalling in PCC [36]. Prior MRS studies have yielded heterogeneous results [13,14,15,16] but most converge on reduced NAA. By applying rigorous correction and normative comparisons, our findings strengthen the case for persistent neurochemical disturbance in PCC. Whether these changes reflect persistent inflammatory activity, residual neuronal stress or longer-term compensatory changes remains unclear.

However, none of these alterations correlated robustly with cognition or symptom burden after correction. Nominal associations suggested links between Glx, Cho, and perceived cognitive change, but these did not survive adjustment. The absence of strong brain–behaviour relationships raises the possibility that measurable neural alterations may not directly map onto cognitive deficits at the individual level or that symptoms are shaped by interacting systemic, immune, and psychological factors. Similarities can be drawn here with functional cognitive disorders. In both, patients report disabling cognitive symptoms, yet objective deficits are modest and imaging correlates elusive [37]. This comparison does not seek to state unequivocally that PCC is a functional disorder but rather that our results support models in which PCC cognitive issues arise from the potential contribution of functional cognitive processes interacting with subtle neurobiological changes.

A further important interpretive consideration is the degree to which comorbid anxiety and depression may contribute independently to the cognitive profile observed. The cohort demonstrated moderate-to-severe symptom burden on both the PHQ-9 (mean 14.0) and GAD-7 (mean 10.8), and there is an extensive literature demonstrating that depression and anxiety independently impair attention, working memory, and verbal fluency—precisely the domains most affected in this cohort. That said, formal analyses of screening-measure–cognition associations did not survive correction for multiple comparisons. There was a nominal association between PHQ-9 and cognitive performance, and this may be consistent with a contributory psychological effect. Disentangling a direct neurobiological effect of SARS-CoV-2 from the broader psychological sequelae of serious illness, prolonged uncertainty, and persistent symptoms remains methodologically challenging in cross-sectional studies. Future longitudinal designs should incorporate pre-specified adjustment for affective comorbidity, and intervention studies targeting mood may help clarify the extent to which cognitive difficulties in PCC are modifiable through psychological treatment.

The study has several strengths, including its multimodal design, rigorous statistical correction, and long follow-up interval. Nevertheless, several limitations should be considered. The sample size was modest, and the study design was exploratory, increasing the risk of type I and type II error across multiple imaging domains. A contemporaneously acquired healthy control group was not available for MRS analysis, and comparisons were therefore made against pooled normative reference datasets that differed in acquisition parameters, processing pipelines, and demographic characteristics. These differences may introduce systematic bias, and MRS findings should therefore be interpreted cautiously. In addition, the cross-sectional design precludes causal inference, and pre-infection baseline data were not available. Vaccination status was not systematically recorded across all participants, as recruitment spanned a period of evolving vaccine availability throughout the pandemic; this represents a further limitation, as vaccination history may independently influence neurobiological and cognitive outcomes in PCC and may account for some of the heterogeneity observed. Similarly, the heterogeneous time from infection to assessment (mean 733 days, SD 269) may have introduced variability in both neuroimaging and neuropsychological findings that was only partially addressed through statistical covariation. The modest sample size precluded adequately powered subgroup analyses stratified by age group and biological sex, both of which are known moderators of cognitive trajectories and neuroimaging metrics; such analyses remain important targets for future larger-scale studies.

Clinically, our findings validate patients’ experiences of attentional and working memory difficulties, while cautioning against over-interpretation of isolated imaging abnormalities. The robust MRS signal raises the possibility that neurometabolite ratios could serve as biomarkers of disease activity or treatment response. However, the interpretation of MRS findings is constrained by the use of pooled normative datasets, which may introduce cohort variability. The lack of direct brain–behaviour correlations underscores the importance of holistic clinical care, addressing fatigue, mood, and functional factors alongside any underlying neuropathology.

Future research should focus on longitudinal multimodal cohorts, ideally with pre-infection baselines. Higher-field MRS and advanced network analyses may further illuminate subtle pathophysiology. Crucially, integration of imaging with detailed symptom profiling—including fatigue, mood, and sleep—will be needed to disentangle the complex interplay of biological and psychosocial drivers.

## 5. Conclusions

We demonstrate selective cognitive impairment and neurometabolic abnormalities in PCC, in the absence of widespread structural changes or clear imaging–cognition correlations. These findings suggest that “brain fog” in PCC likely reflects a constellation of distributed, low-grade neural alterations which interact with psychosocial and functional cognitive processes, rather than a discrete set of structural, neurochemical or connectivity lesions. Recognition of this complexity will be key to advancing both mechanistic understanding and clinical management strategies. Overall, these findings should be viewed as hypothesis-generating and require replication in larger, longitudinal cohorts with matched control data.

## Figures and Tables

**Table 1:** Summary of MRS findings in post-COVID-19 studies.

Study	MRS Region	NAA/Cr	Cho/Cr	mI/Cr	Glx/Cr	Lac/Cr	Notes
**Rapalino 2021** [13]	Corona radiata	↓	↑	↑	↑	↑	*n* = 3
**Poletti 2021** [17]	Anterior cingulate cortex	n/a	n/a	n/a	n/a	n/a	*n* = 49; glutathione only reported.
**Reda 2022** [14]	Unclear; possibly left and right frontal white matter	↓	n/s	n/s	n/s	↑	*n* = 67
**Ernst 2023** [15]	Anterior cingulate cortex (GM)	↓	n/s	↓	n/s	n/a	*n* = 29 vs. controls; two areas examined.
	Frontal white matter	↓	n/s	n/s	↓	n/a	
**Pajuelo 2024** [16]	Corpus callosum (splenium)	n/s	↑	n/s	n/a	n/a	*n* = 55 vs. 21 controls; may not report values as/Cr.
**Thapaliya 2024** [18]	Posterior cingulate cortex	n/a	n/a	n/s	n/s	n/s	Alternative metabolite reporting approach (see study for methodology.)

Cho/Cr = choline to creatine ratio; GM = grey matter; Glx/Cr = glutamate–glutamine to creatine ratio; Lac/Cr = lactate to creatine ratio; mI/Cr = myo-inositol to creatine ratio; MRS = magnetic resonance spectroscopy; NAA/Cr = N-acetylaspartate to creatine ratio; n/a = not assessed; n/s = non-significant difference; *n* = sample number; ↑ = increased relative to controls or normative values; ↓ = decreased relative to controls or normative values.

**Table 2:** Participant Demographics and Characteristics.

Characteristics	Value
Mean age, (years)	58.3 ± 7.9
Sex, n (%)	11 (38%) male; 18 (62%) female
Time from COVID-19 diagnosis to imaging, (days)	732.9 ± 268.9
Radiological evidence of pneumonitis, n (%)	16 (55%)
Age distribution, n (%)	
<40 years	1 (4%)
40–49 years	5 (17%)
50–59 years	9 (31%)
60–69 years	12 (41%)
≥70 years	2 (7%)
Age range (years)	38–80 yrs

Values are presented as mean ± standard deviation or number (n) and percentage (%), unless otherwise stated.

**Table 3:** Group Neuropsychological Test Battery Performance (z-scores).

Test	Mean z-Score ± SD (n)	% Impaired (z < −1.5) (n)
WTAR	0.51 ± 0.68 (16)	0% (0)
Trails A	−0.38 ± 1.46 (24)	17% (4)
Trails B	0.02 ± 1.27 (24)	12% (3)
CVLT Trails 1–5	−0.15 ± 1.37 (24)	17% (4)
TEA (ECD)	−0.93 ± 0.81 (23)	30% (7)
Digit Span Forwards	−1.12 ± 0.68 (21)	33% (7)
Digit Span Backwards	−1.03 ± 0.62 (21)	24% (5)
Verbal Fluency—Letter	−0.47 ± 1.06 (21)	19% (4)
Verbal Fluency—Category	−0.79 ± 1.28 (20)	30% (6)
Rey—Immediate Recall	−0.21 ± 1.28 (11)	18% (2)
Rey—Delayed Recall	−0.61 ± 1.64 (11)	45% (5)

Impairment is defined as z-score < −1.5. Values are mean z-score, standard deviation and number of valid cases (n). WTAR, Wechsler Test of Adult Reading; Trails, Trail Making Test; CVLT, California Verbal Learning Test; TEA (ECD), Test of Everyday Attention—Elevator Counting with Distraction; Rey, Rey–Osterrieth Complex Figure Test.

**Table 4:** Baseline Screening Scores (n = 30).

Measure	Mean ± SD
EQ-5D VAS	51.1 ± 21.4
FACIT-4	19.8 ± 11.7
CCI	62.4 ± 18.6
GAD-7	10.8 ± 6.4
TSQ	3.5 ± 3.4
PHQ-9	14.0 ± 7.2
CORE-10	16.3 ± 8.6

Values are mean (standard deviation). EQ-5D VAS, EuroQol 5-Dimension visual analogue scale 0–100, higher scores indicate better health-related quality of life; FACIT-4, Functional Assessment of Chronic Illness Therapy–Fatigue Scale; scores range from 0 to 52, with higher scores indicating less fatigue; CCI, Cognitive Change Index (self-report version), scores range from 20 to 100, with higher scores indicating greater perceived cognitive decline; GAD-7, Generalised Anxiety Disorder-7 scale, scores range from 0 to 21, with higher scores indicating greater anxiety severity; TSQ, Trauma Screening Questionnaire, 10-item yes/no screening tool for PTSD, with scores ≥ 6 indicating probable PTSD; PHQ-9, Patient Health Questionnaire-9, scores range from 0 to 27, with higher scores indicating greater depression severity; CORE-10, Clinical Outcomes in Routine Evaluation-10, scores range from 0 to 40, with higher scores indicating greater psychological distress.

**Table 5:** Hypothalamus volume (ANCOVA adjusted for eTIV and sex).

Hypothalamic Region	Group Difference (mm^3^)	95% CI Low	95% CI High	*p*-Value	FDR-Corrected *p*-Value
left posterior	−12.45	−19.78	−5.11	*p* = 0.001	***p*** **= 0.047**
whole left	−17.85	−33.84	−1.84	*p* = 0.029	*p* = 0.544
right posterior	−8.32	−16.76	−0.12	*p* = 0.053	*p* = 0.660
whole right	−8.82	−29.78	12.14	*p* = 0.402	*p* = 1.000
left anterior-inferior	−1.23	−2.88	0.43	*p* = 0.143	*p* = 1.000
left anterior-superior	−0.53	−2.30	1.25	*p* = 0.553	*p* = 1.000
left tubular inferior	−0.51	−7.92	6.90	*p* = 0.890	*p* = 1.000
left tubular superior	−3.15	−8.81	2.52	*p* = 0.270	*p* = 1.000
right anterior-inferior	−1.07	−3.12	0.99	*p* = 0.301	*p* = 1.000
right anterior-superior	−0.83	−3.46	1.79	*p* = 0.526	*p* = 1.000
right tubular inferior	5.12	−3.36	13.60	*p* = 0.231	*p* = 1.000
right tubular superior	−3.72	−10.77	3.33	*p* = 0.295	*p* = 1.000

Volumes are means in mm^3^; CI = confidence interval; eTIV = estimated total intracranial volume; FDR = false discovery rate; mm^3^ = cubic millimetres; *p* = *p*-value. Significant corrected *p*-values (*p* < 0.05) are highlighted in bold.

**Table 6:** Connectivity results.

Connection/Cluster	Statistic	*p*-Value	FDR-Corrected *p*
Cluster 4/171 (overall)	*F*(2,51) = 8.01	*p* < 0.001	***p*** **= 0.030**
sLOC r—SPL l	*T*(52) = 3.66	*p* < 0.001	***p*** **= 0.018**
sLOC l—SPL r	*T*(52) = 3.54	*p* < 0.001	***p*** **= 0.019**
sLOC l—SPL l	*T*(52) = 2.96	*p* = 0.005	*p* = 0.070
sLOC r—SPL r	*T*(52) = 2.75	*p* = 0.008	*p* = 0.135

Connection/Cluster lists either the overall network cluster or the individual edges (connections) between brain regions within that cluster. Statistic shows the test used (F = cluster-level ANOVA; T = edge-level *t*-test) with degrees of freedom. Mean difference represents the group difference in connectivity strength (if applicable). *p*-value is the uncorrected probability of the observed effect. FDR-corrected *p* is the *p*-value after false discovery rate correction for multiple comparisons (Benjamini–Yekutieli method). Significant corrected *p*-values (*p* < 0.05) are highlighted in bold. sLOC refers to the lateral occipital cortex, superior division, and SPL refers to the superior parietal lobule. The letters l and r indicate the left and right hemispheres, respectively.

**Table 7:** Magnetic Resonance Spectroscopy Results.

Metabolite	Adjusted Mean	Reference Mean	Difference	Statistic	*p* Value	FDR-Corrected *p*
Cho/Cr	0.429	0.360	0.069	T(28) = 2.90	*p* = 0.007	***p*** **= 0.020**
NAA/Cr	1.293	1.540	−0.247	T(28) = −3.96	*p* < 0.001	***p*** **= 0.002**
Glx/Cr	1.687	1.030	0.657	T(23) = 5.10	*p* < 0.001	***p*** **< 0.001**
mI/Cr	1.066	0.900	0.166	T(28) = 2.41	*p* = 0.023	***p*** **= 0.048**

Metabolite lists each neurochemical measured in the MRS analysis. Cho/Cr, choline-to-creatine ratio (marker of membrane turnover/inflammation); NAA/Cr, N-acetylaspartate-to-creatine ratio (Neuronal integrity); Glx/Cr, glutamate–glutamine-complex-to-creatine ratio (metabolic activity/Glutamatergic activity); mI/Cr, myo-inositol-to-creatine ratio (marker of glial activation); Statistic reports the test used to compare the adjusted metabolite mean against the reference value, shown as a t-statistic with the associated degrees of freedom. FDR-corrected *p* provides the *p*-value after controlling for multiple comparisons using the Benjamini–Yekutieli false discovery rate procedure. Significant corrected *p*-values (*p* < 0.05) are highlighted in bold.

## Data Availability

The raw data supporting the conclusions of this article will be made available by the authors on request.

## Supplementary Materials

The following supporting information can be downloaded at: https://www.mdpi.com/article/10.3390/jcm15114241/s1, Supplementary Table S1: Bilateral volumetric comparison between ACDC and control participants, Supplementary Table S2: White matter lesion comparison ACDC and control participants.
