# Supplementary material for: Assessing Cognitive Deterioration After COVID-19 Infection (The ACDC Study): An Exploratory Multimodal Neuroimaging Study
Source: J Clin Med. 2026 May 30;15(11):4241. doi: 10.3390/jcm15114241 (PMC13257671; doi:10.3390/jcm15114241)
Supplement: Supplementary file 1 [file jcm-15-04241-s001.zip › jcm-4315047-supplementary.pdf]

Supplementary Table S1. Bilateral volumetric comparison between ACDC and control participants

Brain Region	ACDC Mean Volume (mm³) Left	Control Mean Volume (mm³) Left	P Value	FDR Left	ACDC Mean Volume (mm³) Right	Control Mean Volume (mm³) Right	P Value	FDR Right
Bankssts	2111	2045	0.217	0.713	1873	1870	0.495	0.839
Caudal anterior cingulate	1504	1522	0.940	0.969	1957	1891	0.305	0.648
Caudal middle frontal	5152	5654	**0.015**	0.491	5008	5295	0.211	0.553
Cuneus	2793	2833	0.853	0.906	3059	3071	0.600	0.839
Entorhinal	1887	1819	0.167	0.713	1859	1667	**0.011**	0.337
Fusiform	8582	8800	0.824	0.904	8201	8318	0.848	0.945
Inferior parietal	10565	9971	**0.029**	0.491	12506	11944	**0.040**	0.337
Inferior temporal	9415	9928	0.295	0.713	8919	9102	0.972	0.979
Isthmus cingulate	2420	2523	0.320	0.713	2147	2302	0.123	0.511
Lateral occipital	11104	10858	0.104	0.713	11349	10924	**0.034**	0.337
Lateral orbitofrontal	6620	6798	0.667	0.904	6608	6721	0.890	0.945
Lingual	5980	5866	0.273	0.713	6409	6354	0.521	0.839
Medial orbitofrontal	4391	4518	0.692	0.904	4970	4966	0.350	0.663

**Brain Region**	**ACDC Mean Volume (mm³) Left**	**Control Mean Volume (mm³) Left**	**P Value**	**FDR Left**	**ACDC Mean Volume (mm³) Right**	**Control Mean Volume (mm³) Right**	**P Value**	**FDR Right**
Middle temporal	9359	9242	0.190	0.713	10319	10494	0.856	0.945
Parahippocampal	1998	1958	0.253	0.713	1863	1855	0.568	0.839
Paracentral	3058	3027	0.280	0.713	3520	3354	**0.023**	0.337
Pars opercularis	3968	4099	0.634	0.904	3431	3447	0.786	0.945
Pars orbitalis	1987	2042	0.797	0.904	2444	2529	0.602	0.839
Pars triangularis	3065	3072	0.635	0.904	3534	3757	0.269	0.634
Pericalcarine	1759	1774	0.764	0.904	2024	2188	0.280	0.634
Postcentral	8227	8328	0.799	0.904	7944	7812	0.142	0.511
Posterior cingulate	2672	2691	0.777	0.904	2771	2909	0.351	0.663
Precentral	11635	11855	0.969	0.969	11457	11616	0.842	0.945
Precuneus	8682	8578	0.214	0.713	8916	8982	0.617	0.839
Rostral anterior cingulate	2294	2367	0.795	0.904	1902	1802	0.126	0.511
Rostral middle frontal	12768	12718	0.336	0.713	13485	13558	0.526	0.839
Superior frontal	18867	19384	0.748	0.904	17935	18771	0.167	0.511

**Brain Region**	**ACDC Mean Volume (mm³) Left**	**Control Mean Volume (mm³) Left**	**P Value**	**FDR Left**	**ACDC Mean Volume (mm³) Right**	**Control Mean Volume (mm³) Right**	**P Value**	**FDR Right**
Superior parietal	11818	11779	0.428	0.766	11597	11376	0.176	0.511
Superior temporal	10357	10995	0.049	0.558	10048	10544	0.170	0.511
Supramarginal	9613	10049	0.463	0.787	8471	8961	0.180	0.511
Frontal pole	858	929	0.151	0.713	1042	1133	0.122	0.511
Temporal pole	2433	2556	0.405	0.765	2451	2493	0.882	0.945
Transverse temporal	1003	1073	0.229	0.713	769	791	0.754	0.945
Insula	6459	6453	0.380	0.761	6110	6216	0.979	0.979

Cortical regions are defined using the Desikan–Killiany atlas. **Brain Region** refers to the anatomical parcel listed. **ACDC Mean Volume (mm³) Left** and **ACDC Mean Volume (mm³) Right** indicate mean regional volumes (mm³) in the ACDC cohort for the left and right hemispheres, respectively. **Control Mean Volume (mm³) Left** and **Control Mean Volume (mm³) Right** present the corresponding control group means. **P Value** denotes the uncorrected p-value from independent-samples t-tests comparing ACDC and control volumes for each hemisphere. **FDR Left** and **FDR Right** denote false discovery rate (FDR)–adjusted p-values (Benjamini-Yekutieli method) for left- and right-hemisphere comparisons, respectively, controlling for multiple comparisons.

Supplementary Table S2. White matter lesion comparison ACDC and control participants

Lesion Metric	ACDC Mean	ACDC Standard deviation	Control Mean	Control Standard Deviation	P-Value
Lesion volume (ml)	2.89	5.82	3.13	6.09	0.8505
Number of lesions	7.71	5.50	7.39	5.53	0.7673

White matter lesion metrics include total lesion volume and number of discrete lesions, reported as mean ± standard deviation (SD) per group. Group comparisons were performed using the Wilcoxon rank-sum test due to non-normal distribution of the data. No significant group differences were found for either metric ($p > 0.05$).
